# Supplementary material for: Predictors of functional improvement in the short term after MitraClip implantation in patients with secondary mitral regurgitation
Source: PLoS One. 2020 May 28;15(5):e0232817. doi: 10.1371/journal.pone.0232817 (PMC7255600; doi:10.1371/journal.pone.0232817)
Supplement: S1 Table — (DOCX) [file pone.0232817.s001.docx]

**S1 Table: Echocardiographic predictors of ∆6MWD four weeks after MitraClip implantation in the multiple linear regression model.**

|  | B | SE | β | t | p |
| --- | --- | --- | --- | --- | --- |
| 6MWD at baseline | -0.3 | 0.1 | -0.46 | -3.56 | **0.001** |
| Age | -1.1 | 1.4 | -0.12 | -0.84 | 0.406 |
| MR grade | 17.6 | 20.5 | 0.11 | 0.86 | 0.396 |
| LVEF | 0.9 | 0.8 | 0.17 | 1.14 | 0.258 |
| LVEDD | 1.8 | 1.6 | 0.24 | 1.11 | 0.273 |
| LAVI | -0.1 | 0.2 | -0.03 | -0.21 | 0.833 |
| LVMI | -0.3 | 0.3 | -0.18 | -1.06 | 0.292 |
| R | 0.476 | | | | |
| R^2^ | 0.226 | | | | |
| Adjusted R^2^ | 0.134 | | | | |
| F | (7, 59) = 2.463 | | | | |
| p | 0.028 | | | | |
| n | 67 | | | | |

Results on the predictors are reported as coefficient B, standard error SE, standardized coefficient β, t-statistic t and p-value. Overall model characteristics are reported as multiple correlation coefficient R, coefficient of determination R^2^ and F-ratio F.

LAVI, left atrial volume index; LVEDD, left ventricular end diastolic diameter; LVEF, left ventricular ejection fraction; LVMI, left ventricular mass index; MR, mitral regurgitation; SE, standard error; 6MWD, six-minute walk distance.
