# Supplementary material for: Predictors of functional improvement in the short term after MitraClip implantation in patients with secondary mitral regurgitation
Source: PLoS One. 2020 May 28;15(5):e0232817. doi: 10.1371/journal.pone.0232817 (PMC7255600; doi:10.1371/journal.pone.0232817)
Supplement: S2 Table — (DOCX) [file pone.0232817.s002.docx]

S2 Table: Correlation between post-procedural mitral valve mean pressure gradient and ∆6MWD.

|  | B | SE | β | t | p |
| --- | --- | --- | --- | --- | --- |
| 6MWD at baseline | -0.1 | 0.1 | -0.23 | -1.79 | 0.079 |
| Age | -1.2 | 1.1 | -0.14 | -1.10 | 0.274 |
| MR grade | 23.3 | 15.8 | 0.17 | 1.47 | 0.146 |
| MV mean pressure gradient 4 weeks after MCI, mmHg | -9.2 | 4.0 | -0.27 | -2.32 | **0.023** |
| R | 0.411 | | | | |
| R^2^ | 0.169 | | | | |
| Adjusted R^2^ | 0.119 | | | | |
| F | (4, 66) = 3.363 | | | | |
| p | 0.014 | | | | |
| n | 71 | | | | |

Results of multiple linear regression are reported as coefficient B, standard error SE, standardized coefficient β, t-statistic t and p-value. Overall model characteristics are reported as multiple correlation coefficient R, coefficient of determination R^2^ and F-ratio F.

MCI, MitraClip implantation; MR, mitral regurgitation; MV, mitral valve; SE, standard error; 6MWD, six-minute walk distance.
