# Supplementary material for: Predictors of functional improvement in the short term after MitraClip implantation in patients with secondary mitral regurgitation
Source: PLoS One. 2020 May 28;15(5):e0232817. doi: 10.1371/journal.pone.0232817 (PMC7255600; doi:10.1371/journal.pone.0232817)
Supplement: Attachment — Submitted filename: rebuttal_letter.docx [file pone.0232817.s004.docx]

Rebuttal Letter

# Reviewer 1

We gratefully appreciate that Reviewer 1 acknowledges our investigation of the functional outcome after MitraClip implantation. We are also very thankful for the valuable comments which we would like to address as follows.

*The Reviewer expresses that the trial’s clinical impact and its use for patient selection is limited because it did not investigate hard endpoints.*

We clearly agree with the Reviewer’s opinion that patient selection for MitraClip implantation should primarily be based on hard endpoints like mortality. Nevertheless, in our clinical experience, patients who consider undergoing MitraClip expect symptom relief and improvement in their functional status, which is why clinical endpoints are increasingly acknowledged as relevant outcome parameters. Therefore, in our opinion, short-term improvement in functional capacity as expressed by an increase in 6MWT is still an important aspect of the outcome of MitraClip therapy. This is especially true for the collective of patients with secondary mitral regurgitation, whose prognosis is often significantly limited by the severe comorbidities. Yet, we acknowledge that while our study adds new hypothesis-generating aspects, guidance for patient selection cannot be given solely on the basis of our results. In our revised manuscript, this is clarified in the discussion section.

*The Reviewer asks for details on the inclusion process and possible selection bias, as he perceives a discrepancy between the number of included patients and the length of the inclusion period.*

To more clearly demonstrate the inclusion process of our study, we added a detailed flowchart to the methods section of our revised manuscript which explicitly states the total number of patients who underwent MitraClip therapy at our center during the inclusion period. The most frequent reason for exclusion was MR of primary origin. As in many other MitraClip centres, the quantity of procedures increased over the years, clearly reflecting the growing clinical acceptance of this therapeutic modality. Due to the retrospective nature of our study, we do not have information on the reasons for patients not completing the follow-up examinations. Consequently, attrition bias cannot be ruled out when interpreting our results. Therefore, in the revised manuscript, we added this fact as a limitation in the discussion section.

*The Reviewer asks for data on NYHA class and mortality at later timepoints and their correlation with short-term increase in 6MWD.*

We agree with the Reviewer that the correlation between improvement in 6MWD and long-term functional class is an important aspect and are thankful for this valuable comment. Therefore, in the revised manuscript, we included data on NYHA functional class twelve months after the procedure which is available for the majority of patients. Additionally, further statistical analysis revealed a moderate correlation between postprocedural 6MWD and NYHA functional class both four weeks and twelve months after the procedure.

Because our study design was dedicated to functional outcome after MitraClip therapy, we did not evaluate mortality endpoints.

*The Reviewer requests an analysis of the interaction between post-procedural MVPG and improvement in 6MWD.*

We are grateful for this excellent remark. In the revised manuscript, we conducted an additional analysis, revealing a negative correlation between post-procedural MVPG and increase in 6MWD. This indeed may play an important role in the observed gender-specific differences, which is outlined in the discussion section of the revised manuscript.

*The Reviewer states that the trial’s cohort, although limited to patients with secondary MR, is heterogenous regarding the pathomechanism of MR.*

The Reviewer is right that our study population comprises numerous entities of secondary MR and underlying structural heart disease, reflecting the patient collective treated with MitraClip implantation. In the revised manuscript, this is clarified in the discussion section.

*The Reviewer requests further clarification of the aspect that while female gender predicted worse improvement in 6MWD, NYHA functional class is not different between male and female patients. Additionally, the Reviewer asks if assessment of 6MWD in our study might have been subject to a learning effect.*

Indeed, while female gender was identified as an independent predictor of less improvement in 6MWD, NYHA class was not significantly different between male and female patients. This is likely explained by the limitations of the NYHA classification: It is subjective and exhibits a rather low reproducibility rate. In contrast, the 6MWT is an objective measure of functional performance with high reproducibility in patients with heart failure. Therefore, we consider the 6MWT to be superior in detecting differences in functional outcome. In the revised manuscript, this is clarified in the discussion section.

The Reviewer is right that 6MWT exhibits a training effect when conducted repeatedly during one day or one week. However, as our study population only performed 6MWT twice and four weeks apart and considering the immobilization during the hospital stay, we consider a significant training to be unlikely. Still, it cannot be completely ruled out when interpreting the results.

*The Reviewer requests data on medication before and after MitraClip therapy, as this might also influence functional capacity.*

We agree that a change in medication, particularly diuretics intake, might have an impact on functional capacity. Therefore, in the revised manuscript, we included data on medication at baseline and four weeks after the procedure, which did not change significantly. Also, medication four weeks after MitraClip therapy did not differ between men and women.

*The reviewer suggests that the data on NTproBNP should be presented as median and percentiles.*

We are thankful for this justified remark. In the revised manuscript, NTproBNP is presented as median and percentiles.

*The reviewer asks for data of quality of life questionnaires.*

We clearly agree with the reviewer that quality of life questionnaires are very important tools for further investigation of functional outcome. As our study was retrospective and quality of life questionnaires were not part of our routine clinical care, we cannot provide these additional data. In our revised manuscript, this aspect was added as a limitation in the discussion section.

# Reviewer 2

We are thankful that the Reviewer finds our manuscript interesting and its findings hypothesis-generating. We also appreciate the useful comments to which we would like to respond as follows:

*The Reviewer suggests adding age and baseline echocardiographic parameters such as MR grade as covariates in the linear regression model.*

We appreciate this valuable recommendation. In the revised manuscript, age and baseline MR grade were added as covariates in the linear regression model.

*The Reviewer recommends performing sensitivity or subgroup analyses.*

We acknowledge that conducting sensitivity analysis would be useful to further confirm the validity of the identified predictors. However, this possibility is restricted by the sample size. Performing the regression model on a subgroup (e.g. female patients) would reduce the sample size to such an extent that overfitting of the model occurs, leading to invalid results. We added this aspect in the limitations section of the revised manuscript.

*The Reviewer asks for an additional regression model which includes echocardiographic parameters, particularly parameters of diastolic dysfunction.*

We are grateful for this recommendation. In the revised manuscript, we conducted an additional regression model which includes several echocardiographic parameters. No independent echocardiographic predictor was identified. As a measure for diastolic dysfunction, left ventricular mass index and left atrial volume index were analyzed. As tissue doppler imaging was not part of our clinical routine follow up, we are not able to provide more specific parameters of diastolic function.

*The Reviewer states that our discussion cannot draw conclusions on device success rate as patients who failed to implant MitraClip or did not complete follow-up were omitted from analysis.*

We fully agree that our study is not able to draw conclusions on device success due to its study design. Therefore, we omitted this sentence in the revised manuscript.

*The Reviewer expresses that the impact of the study is limited by the sample size and asks for further details on the number of excluded patients.*

To elucidate the inclusion process, we included a study flow chart to the methods section of the revised manuscript, depicting the number of patients who underwent MitraClip implantation at our center during the inclusion period as well as the number of patients who were excluded from analysis. Exclusion because of failure to implant a clip was rare, occurring only in six patients. Patients without a 6MWT were excluded in the intent of a complete case analysis. As we do not have information on the reasons why patient did not perform 6MWT, we agree that attrition bias cannot be ruled out when interpreting our results. In the revised manuscript, this was added as a limitation in the discussion section.

As mentioned in the discussion section of our manuscript, we agree that the sample size could be seen as a limitation to our study. While its results need to be validated in a larger cohort, our trial may aid to improve patient selection and generate new hypotheses on the mechanisms determining therapeutic response to MitraClip implantation.

*The Reviewer asks for definition of MR grade in the methods section.*

Corresponding to this comment, we detailed MR grading in the methods section.
