# Supplementary material for: Predictors of functional improvement in the short term after MitraClip implantation in patients with secondary mitral regurgitation
Source: PLoS One. 2020 May 28;15(5):e0232817. doi: 10.1371/journal.pone.0232817 (PMC7255600; doi:10.1371/journal.pone.0232817)
Supplement: Attachment — Submitted filename: response_to_reviewers.docx [file pone.0232817.s005.docx]

Response to Reviewers

# Reviewer 4

We thank Reviewer 4 for thoroughly reviewing our manuscript and appreciate the valuable comments which we would like to address as follows.

*The Reviewer expresses that the observed gender-specific difference in functional outcome might in part be caused by differences in the baseline characteristics between male and female patients. For further clarification, the Reviewer recommends adding baseline parameters with observed gender-specific differences to the regression model.*

We are grateful for this excellent remark. In the revised manuscript, we added baseline LVEDD, preserved LVEF and NYHA functional class as variables to the regression model. After correcting for these parameters, female gender remains a significant predictor of 6MWD improvement, confirming our findings. Yet, we fully agree with the Reviewer’s opinion that the reasons for the observed gender-related difference are multifactorial and may in part be explained by differences in baseline characteristics, especially in LV geometry and systolic function. However, given similar findings in the analysis of the GRASP and TRAMI registry, these gender-specific differences in baseline characteristics do not occur exclusively in our patient collective, but appear to be inherent in the population treated with TMVR. In the revised manuscript, these considerations were added to the discussion section.

*The Reviewer asks for data on 6MWD improvement at later timepoints.*

We agree with the Reviewer that, although short-term 6MWD strongly correlated with long-term NYHA class in our cohort, data on 6MWD at later timepoints could give further insight into determinants of functional outcome. However, as evaluation of long-term 6MWD was not part of our routine follow-up, we do not have sufficient data for valid statistical analysis. In the revised manuscript, this aspect was added as a limitation in the discussion section.

*The Reviewer suggests a consistent use of either “6MWD” or “6MWT” as an abbreviation for the six-minute walk distance.*

In the previous version of our manuscript, the abbreviation “6MWT” was used when referring to the walk test itself while “6MWD” referred to the result of the walk test. We clearly agree with the reviewer that this might confuse readers. Therefore, in the revised manuscript, we now consistently use the abbreviation “6MWD”.
